# Implantable mechanical circulatory support in The Netherlands

**DOI:** 10.1007/s12471-023-01782-3

**Published:** 2023-04-18

**Authors:** Jaap R. Lahpor

**Affiliations:** 1407 Houston Cr, Kanata, Ontario Canada

The article *Left ventricular assist device implantation and clinical outcomes in the Netherlands *in this issue by Damman et al. marks a journey that started 30 years ago with the first implantation of a pulsatile implantable left ventricular assist device (LVAD) as bridge to heart transplantation (BTT) [1]. Following the start of heart transplantations in the Netherlands a decade earlier, it had become a successful treatment for patients with advanced heart failure with reduced ejection fraction (HFrEF). However, on average one in five heart transplant candidates on the waiting list did not survive before a donor heart became available. Mechanical circulatory support (MCS) was the best option for these patients to stay alive during the waiting process.

The first pulsatile implantable LVADs were voluminous, noisy, and technically vulnerable devices. They were associated with serious complications such as infections, bleeding issues, pump thrombosis and thrombo-embolic events. Nevertheless, the first experience with these devices in Utrecht showed promising outcomes by successfully supporting patients until heart transplantation. However, adverse events as well as technical dysfunction were limiting factors for survival beyond 6–12 months of support. This became evident in a study randomising end-stage heart failure patients for either MCS or optimal medical treatment (REMATCH trial) [2]. This study showed no survival difference for both treatment groups with only few survivors after 2 years.

In the Netherlands, the introduction in 2006 of a 2nd generation non-pulsatile LVAD, the HeartMate 2 axial flow pump, was an important step forward. This device proved to be technically more reliable, resulting in a 2-year survival around 70% paired with a better quality of life [3]. Adverse events, however, remained a major issue with 19% stroke rates, 12% pump thrombosis and 52% bleeding complications [3]. Nevertheless, this improved 2‑year survival was the impetus to other device strategies such as destination therapy for non-eligible heart transplant candidates (DT) and bridge to decision (BTD). These strategies definitely became a topic after the introduction of the 3rd generation centrifugal devices, HeartMate 3 and HeartWare HVAD, in 2016.

The article of Damman et al. is unique being the first combined outcome report of all Dutch VAD implanting centres.

The HeartMate 3 with almost two-thirds of implants is the most used VAD in their study. It has been claimed that this VAD is more haemocompatible due to its bearing-less, magnetically levitated impeller, thereby reducing stress on blood cells and preventing stasis and blood clot formation in the pump.

The outcomes in the study support that claim reporting a significant lower pump thrombosis and cerebrovascular event rate for the HeartMate 3 compared with the HVAD and HeartMate 2 (0.08 vs 0.21 vs 0.44 and 0.01 vs 0.06 vs 0.02 events/patient-year; Supplementary Table 1) [1]. This is conform major prospective international studies [4, 5]. The HeartMate 3 also scored significantly lower in bleeding event and infection complication rates. However, reading Supplementary Fig. 4 in the study, it is still sobering that only 27% of patients had been event-free after 2 years and 9% after 5 years [1].

Overall survival irrespective of device strategy (BTT, BTD or DT) is comparable with previously mentioned studies [4, 5]. It should be noted though, that in the Momentum 3 trial 1‑year and 2‑year survival rates for the axial flow HeartMate 2 and centrifugal HeartMate 3 did not statistically significantly differ [5]. That only became evident in a follow-up study for the following 3 years in favour of the HeartMate 3 [6]. Looking at the event-free 5‑year survival (free from pump dysfunction and cerebrovascular events), the same study showed the HeartMate 3 being the superior device [6].

In the discussion, the authors indicate the high annual number of VAD implants in the Netherlands in contrast to the United States, where it has dropped by 23.5% in 2021 [7] and where a change in the heart donation allocation system has resulted in a dramatic reduction in number of patients transplanted on devices [8, 9]. This came paired with a modest increase (4.3%) in total number of heart transplantations (https://unos.org>news >2022). The competing outcome statistics show that presently around 50% of US patients on devices may expect to receive a heart transplant within 3 years [8]. In the Netherlands, this is only 20% in 3 years and 45% after 5 years (Fig. S1) [1]. Low number of heart donations and long waiting times are the main responsible factors, not—in all probability—the national waiting list allocation. One may hope that the change in organ donor registration in 2020 and new preservation and organ harvesting techniques will insure higher heart transplantation rates in the years to come. Nevertheless, there will undoubtably remain an important role for MCS in the future. Hopefully a follow-up study with only the Mag Lev Centrifugal HeartMate 3 LVAD will provide more clarity in this regard.
